# Gut microbiota dysbiosis at the interface of neuropsychiatric disorders and their dermatological comorbidities

**DOI:** 10.1080/19490976.2025.2574934

**Published:** 2025-11-24

**Authors:** Brittany Hawkins, Maddison Montgomery, Gabriela Bokota, Maria Santoyo, Erica Giron, Ahmed Eltokhi

**Affiliations:** a School of Medicine, Mercer University, Columbus, GA, USA; b Department of Biomedical Sciences, School of Medicine, Mercer University, Columbus, GA, USA

**Keywords:** Gut microbiota dysbiosis, neuropsychiatric disorders, dermatological comorbidities, gut–brain–skin axis, probiotics

## Abstract

Neuropsychiatric disorders such as autism spectrum disorder (ASD), generalized anxiety disorder (GAD), major depressive disorder (MDD), and schizophrenia (SZ) frequently co-occur with dermatological conditions, including atopic dermatitis, psoriasis, rosacea, and chronic urticaria. The biologic basis remains incompletely understood. A growing body of evidence implicates gut microbiota dysbiosis as a shared pathogenic factor linking these conditions. This review synthesizes preclinical and clinical findings demonstrating consistent microbial alterations across both neuropsychiatric and dermatologic conditions, including fluctuations in alpha diversity, disrupted Firmicutes/Bacteroidetes ratios, and depletion of short-chain fatty acid (SCFA)–producing taxa such as *Faecalibacterium*, *Roseburia*, and *Eubacterium* species. These microbial shifts parallel elevations in inflammatory mediators such as interleukin-6 (IL-6), tumor necrosis factor-alpha (TNF-α), interleukin-1beta (IL-1β), and interleukin-17 (IL-17), and cause perturbations in amino acid metabolism, altered glutamate-GABA signaling and increased branched-chain amino acids, indicating convergence on immune and metabolic pathways. Experimental rodent studies support the concept by demonstrating that microbiota dysbiosis can both impact psychiatric-like behaviors and cutaneous inflammation. Microbiota-targeted therapies such as probiotics show preliminary efficacy in improving symptoms across both domains. These findings support a gut microbiota–brain–skin axis and suggest that targeting gut dysbiosis may offer an integrated therapeutic approach for neuropsychiatric disorders and their dermatologic comorbidities.

## 1. Introduction

The gut–brain–skin axis is an emerging concept that highlights the complex, intertwined relationships between gastrointestinal (GI) health, nervous system signaling, and skin conditions. The GI tract, which runs from the mouth to the anus, is essential not only for digestion and nutrient absorption but also for supplying the body with key building blocks for systemic functions. Beyond these basics, the GI tract plays a central role in immune defense by protecting against pathogens and regulating immune activity. It also contributes to hormone production that influences appetite, digestion, and insulin release.^1^ While the gut handles defense, metabolism, and hormone signaling, the brain serves as the body's command center. It governs everything from physiological regulation to higher cognitive functions and emotional processing. Cognitive processes like learning, memory, and attention are deeply connected with emotions, moods, and behavior, reflecting how the brain operates as both a thinking and feeling organ.^2^^,^^3^ The skin, often viewed as a simple protective barrier, is in fact a dynamic organ with wide-ranging functions. It supports immune responses through its resident immune cells, regulates temperature and sensory input, and helps prevent water loss. It also plays a key role in vitamin D synthesis and overall homeostasis.^4–6^ Although the gut, brain, and skin are often studied in isolation, these systems do not function independently.

Neuropsychiatric disorders sit at the intersection of neurology and psychiatry, blending cognitive, emotional, and behavioral symptoms. The mid-20th century saw the development of neuropsychiatry as a field in response to overlapping symptoms and shared neurological underpinnings.^7^ Imbalances in neurotransmitters such as Gamma-Aminobutyric Acid (GABA), dopamine, serotonin, and glutamate are common across neuropsychiatric conditions like anxiety and depression, and reinforce the need for an integrated model of brain function.^8^ Despite advances in clinical care, the overall prevalence and disability associated with these conditions have not declined over the past three decades.^9^ A growing body of research indicates that patients with neuropsychiatric disorders frequently present with comorbid dermatological conditions, while individuals with chronic skin diseases often experience psychiatric symptoms. Recent epidemiological studies highlight this bidirectional relationship, with psychiatric comorbidities reported in up to one-third of dermatology patients, and higher rates of dermatological conditions observed among those with psychiatric illness (For recent reviews and meta-analyses, see ^10–13^).

An area of growing scientific interest is the role of gut microbiota in the pathophysiology of neuropsychiatric and dermatological disorders. The gut microbiota is a diverse community of bacteria, viruses, fungi, and archaea colonizing the GI tract from birth and evolving throughout life.^14–16^ These microbes digest complex carbohydrates, producing short-chain fatty acids (SCFA) that fuel colonocytes and reduce inflammation,^17–19^ synthesize key vitamins like K and B,^20^ and shape immune development, maintain tolerance, and preserve epithelial barrier integrity.^21^ Disruption of this microbial balance, or dysbiosis, has been implicated in both neuropsychiatric and dermatological disorders, raising the possibility that the gut microbiota could serve as a shared biological interface connecting these conditions.

In this review, we aim to explore the hypothesis that alterations in gut microbiota may represent a unifying mechanism underlying both brain- and skin-related disorders by synthesizing evidence from both preclinical and clinical studies. To identify recurring patterns and shared mechanisms, we first examine gut microbiota dysbiosis separately in several neuropsychiatric disorders and dermatological conditions. Rather than asserting direct causality, we highlight overlapping mechanisms, converging biomarkers, and shared pathways. Ultimately, we propose that targeting the gut microbiota dysbiosis may offer a novel and integrative therapeutic strategy for simultaneously managing core psychiatric symptoms and their comorbid dermatological manifestations.

## 2. Neuropsychiatric disorders and their associated dermatological comorbidities

Neuropsychiatric disorders are highly complex conditions characterized by diverse clinical manifestations and multifaceted comorbidities. Concurrent dermatological manifestations have gained more attention due to their frequent co-occurrence and potential shared pathophysiological mechanisms. In this section, we focus on four major neuropsychiatric disorders, autism spectrum disorder (ASD), generalized anxiety disorder (GAD), major depressive disorder (MDD), and schizophrenia (SZ), and identify their most commonly reported dermatological comorbidities. We also explore whether there are overlapping dermatological conditions shared across these different neuropsychiatric disorders, which may provide new insights into their interconnected biological underpinnings.

### 2.1. Autism spectrum disorder

ASD is a neurodevelopmental condition characterized by social and communication deficits and repetitive behaviors, affecting approximately 1 in 36 children in the US, with a higher prevalence in males.^22^ While its etiology is multifactorial, encompassing genetic, immunological, and environmental factors, there is increasing recognition of immune dysregulation and gut microbiota imbalance as important contributors.^23^^,^^24^

Dermatological comorbidities such as atopic dermatitis and psoriasis have been reported at higher rates among individuals with ASD. Atopic dermatitis, a chronic inflammatory skin disease, often presents in early childhood, coinciding with ASD onset, and affects up to 20% of children and 10% of adults.^25^ Several studies have reported increased ASD risk in those with atopic dermatitis,^26–28^ with shared immune features including elevated IL-6, IL-17, TNF-*α*, mast cell activation, and vitamin D dysregulation.^29–31^ Psoriasis, a Th17/IL-23-mediated skin disorder, has also been found to occur more frequently in ASD, potentially linked to elevated IL-17A expression observed in both skin and brain tissues.^32^ These findings suggest converging inflammatory pathways that may underlie the co-occurrence of ASD and inflammatory dermatoses.

### 2.2. Schizophrenia

SZ is a chronic psychiatric disorder characterized by delusions, hallucinations, or disorganized speech, alongside negative symptoms such as flat affect or avolition. Diagnostic criteria requires two or more symptoms over one month and functional impairment for at least six months.^33^ Lifetime prevalence ranges from 0.3% to 0.7%, with increased rates in urban areas but no sex-based differences.^33^ Immune dysfunction is increasingly implicated in SZ, with elevated serum levels of IL-6, IL-1β, IL-8, and TNF-*α* being reported.^34–36^ Notably, 60% of individuals later diagnosed with SZ had previous hospitalizations for infections, suggesting blood–brain barrier compromise may contribute to the onset.^37^

Dermatologic comorbidities, particularly psoriasis, are well documented in SZ. Psoriasis is more prevalent in SZ patients, with odds ratios ranging from 1.41 to 1.48 in large-scale studies.^38–40^ These disorders share immunological signatures, including upregulated IL-6 and TNF-*α*. Autoimmune contributions are also suggested by shared HLA polymorphisms^41^ and evidence of disrupted barrier integrity, a possible mechanism linking peripheral inflammation to central pathology.^42^

Associations between SZ and atopic dermatitis are less clear. While one study reported dermatitis in nearly half of SZ patients,^43^ limited awareness of dermatologic conditions may have confounded results. A recent study found higher SZ prevalence in patients with atopic dermatitis,^44^ while two population-based studies reported an inverse association, particularly in severe cases.^45^^,^^46^ Given these conflicting findings, further research is needed to clarify shared pathophysiological mechanisms.

### 2.3. Major depressive disorder

MDD is characterized by a depressive episode lasting at least two weeks, marked by symptoms such as anhedonia, depressed mood, fatigue, weight changes, sleep disturbances, poor concentration, feelings of worthlessness, and suicidal ideation.^33^ Affecting approximately 7% of the US population annually, MDD is most common in young adults and the elderly.^47^ Central to its pathophysiology is monoamine neurotransmitter dysregulation, particularly serotonin, dopamine, norepinephrine, and GABA – alongside mesocortical and mesolimbic pathway alterations.^48^ Additionally, dysregulation of the hypothalamic-pituitary-adrenal (HPA) axis leads to reduced brain-derived neurotrophic factor (BDNF), contributing to stress-induced neuronal dysfunction.^49^^,^^50^ MDD is further characterized by systemic immune activation, with elevated IL-6, IL-1β, TNF-*α*, and IFN-*γ* levels.^51–53^

MDD exhibits bidirectional comorbidity with dermatological conditions, particularly atopic dermatitis and psoriasis, which may exacerbate depressive symptoms through visible disfigurement, pruritus, and social stigma.^54^^,^^55^ Inflammatory overlap is prominent: both disorders exhibit elevated cytokines such as IL-6, IL-1β, and TNF-*α*, reinforcing the hypothesis of a shared immune axis.^51–53^ In one pediatric study, atopic dermatitis was associated with a 1.81-fold increase in depression risk,^56^ while broader population studies found elevated rates of depression and suicidal ideation in severe cases.^57^^,^^58^ Psoriasis is also strongly associated with MDD, with a meta-analysis of over 260,000 patients reporting a 19% prevalence of depression,^59^ and those with MDD were found to be 1.3 times more likely to develop psoriasis.^60^ Proinflammatory cytokines such as IL-6 and TNF-*α*, and their ability to influence corticotropin-releasing hormone (CRH) expression and HPA axis signaling, may contribute to this link.^49^^,^^50^^,^^61^ These findings support a complex interface between neuroendocrine stress responses, immune dysregulation, and cutaneous inflammation in MDD.

### 2.4. Generalized anxiety disorder

GAD is defined by persistent, excessive worry across various domains for a duration of at least six months, accompanied by symptoms such as restlessness, fatigue, difficulty concentrating, irritability, muscle tension, and sleep disturbance.^33^ GAD affects approximately 2.9% of adults annually in the US.^62^ Systemic inflammation has been implicated in GAD, with elevated serum levels of C-Reactive Protein (CRP), IL-1β, IL-6, and IL-17 reported in affected individuals.^63^^,^^64^

Emerging literature highlights a bidirectional relationship between GAD and chronic inflammatory dermatoses, particularly rosacea. Rosacea is a chronic inflammatory skin disorder characterized by recurrent facial erythema, flushing, telangiectasias, papules, pustules, and, in some cases, ocular involvement.^65^ Anxiety disorders, including GAD, are more than twice as prevalent in individuals with rosacea, with one meta-analysis underscoring this elevated risk.^66^ While psychosocial factors may contribute to this comorbidity, overlapping inflammatory signatures, particularly elevations in IL-17 and IL-1β, suggest a shared pathophysiological mechanism.^65^^,^^67^^,^^68^ Although findings regarding IL-6 levels remain inconsistent,^67^^,^^69^^,^^70^ the elevation of Th17-related cytokines across both conditions is well supported. While a direct genetic linkage remains inconclusive,^71^ the convergence of immune and neuroinflammatory pathways supports their mechanistic overlap.^72^^,^^73^

Chronic urticaria (CU), defined as the occurrence of wheals and/or angioedema lasting more than six weeks, is another condition frequently comorbid with GAD. Its pathophysiology involves dermal mast cell activation through autoimmune (IgE/IgG) or non-autoimmune pathways.^74^ Genetic polymorphisms in CU patients are linked to elevated proinflammatory cytokines such as IFN-*γ*, IL-6, IL-17, and TNF.^75^ Psychological stress activates the HPA axis, releasing CRH, IL-1β, TNF-*α*, and neuropeptides like substance *P*, promoting mast cell degranulation.^76^ Multiple studies have reported the high prevalence of anxiety symptoms in individuals with CU, with one study reporting that 29% of patients experienced anxiety symptoms^77^ and another study finding a 50% self-reported anxiety rate among 270 CU patients undergoing treatment.^78^ In contrast, a larger cohort of 1539 patients reported a lower prevalence of 4.7%.^79^ A meta-analysis of 644 CU patients estimated a pooled anxiety prevalence of 30.6%,^80^ and a recent study using the GAD-7 screening tool reported that 41.3% met criteria for GAD.^81^ Although prevalence estimates vary, the consistency of HPA axis involvement, systemic inflammation, and mast cell dysregulation in both CU and GAD suggests a shared biological vulnerability.^63^^,^^74^^,^^82^^,^^83^

In summary, these findings indicate that certain dermatological conditions, particularly atopic dermatitis and psoriasis, are consistently shared across multiple neuropsychiatric disorders, including ASD, MDD, and SZ, while rosacea and CU show stronger links with anxiety-related phenotypes such as GAD. This overlap highlights converging inflammatory and neuroimmune pathways that may underlie both psychiatric and dermatological disease expression.

## 3. Gut microbiota dysbiosis in neuropsychiatric disorders

**Figure 1.**
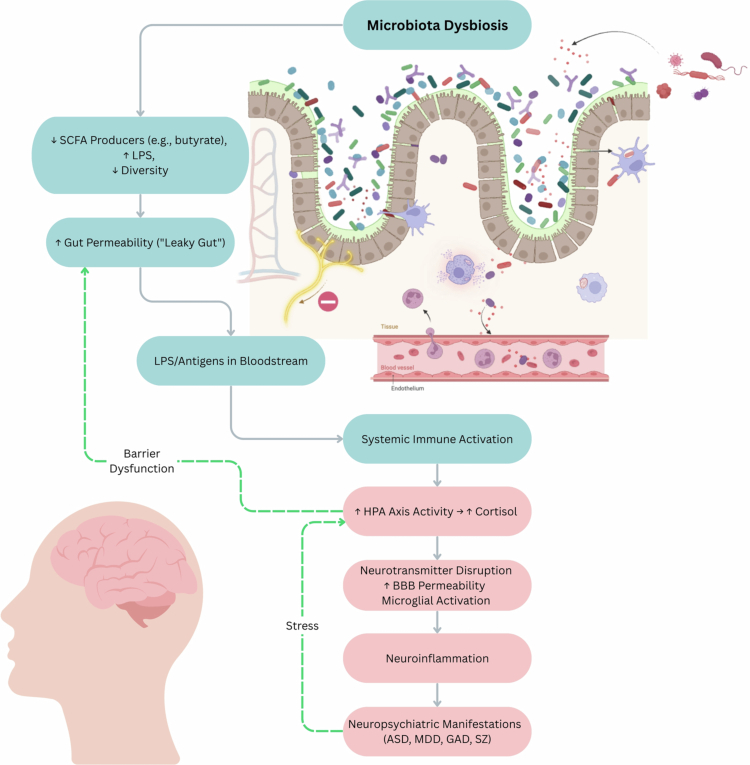
Microbiota dysbiosis and neuropsychiatric manifestations via gut–brain axis disruption. A schematic model illustrating how microbiota dysbiosis contributes to the development of neuropsychiatric conditions through gut–brain axis disruption. Loss of short-chain fatty acid (SCFA) producers (e.g., *Faecalibacterium* and *Roseburia*), expansion of lipopolysaccharide (LPS)-bearing taxa, and decreased microbial diversity contribute to increased intestinal permeability (“leaky gut”) and antigenic translocation into the bloodstream. This initiates systemic immune activation, triggering elevated hypothalamic-pituitary-adrenal (HPA) axis activity and cortisol release. The HPA axis is further activated by the stressors associated with neuropsychiatric conditions. Resulting downstream effects include disruption of neurotransmitter signaling, increased permeability of the blood–brain barrier (BBB), and activation of microglia. This progresses to neuroinflammation implicated in the pathogenesis of autism spectrum disorder (ASD), major depressive disorder (MDD), generalized anxiety disorder (GAD), and schizophrenia (SZ). Green dashed arrows indicate positive feedback mechanisms between peripheral and central stress and barrier systems. Created in BioRender. Hawkins, B. (2025) https://BioRender.com/adla0kf. Final figure graphics made in Canva.

The gut microbiota performs vital physiological roles through complex microbial communities that begin forming at birth and evolve throughout life. In a healthy state, the gut microbiota plays a critical role in modulating immune responses, regulating endocrine signaling, and influencing neurotransmitter synthesis and availability. However, over the lifespan, influences such as diet, medication use, and exposure to environmental pollutants can disrupt this balance, potentially tipping the microbiota toward dysbiosis. Early evidence for gut–brain connections appears even in young children, as reported in toddlers aged 18–27 months, where children exhibiting greater surgency and extraversion traits displayed higher phylogenetic diversity in their gut microbiota.^84^ A growing body of work links microbiota dysbiosis to neuropsychiatric disorders through mechanisms including reduced alpha diversity, altered microbial communities, immunogenic epitopes, microbial toxins, inflammation, and neurotransmitter modulation^85–89^ (Figure 1). Given the high heterogeneity of neuropsychiatric disorders and their frequent co-occurrence with systemic comorbidities, both across and within diagnoses, it is essential to first dissect the shared pathophysiology of their core phenotypes in the context of gut microbiota dysbiosis before attempting to identify common mechanisms with their comorbidities, which may aid in understanding, diagnosing, and treating such disorders.

In Table 1, we compile all available clinical studies reporting gut microbiota dysbiosis in ASD, SZ, MDD, and GAD separately. A review of these studies reveals complex yet overlapping patterns of dysbiosis across major neuropsychiatric disorders. Despite variability, consistent trends of increased or decreased microbial abundance and diversity emerge, indicating the presence of both disorder-specific and shared microbial signatures.

**Table 1.** Clinical studies of gut microbiota dysbiosis in neuropsychiatric disorders.

Reference	Related condition	Study type	Sample size	Microbial alteration	Key findings
[90]	ASD	Comparative Study	58 ASD children (3–16 years old)	↑ Relative Abundance clostridium histolyticum	Children with ASDs have a higher incidence of the Clostridium histolyticum group in their gut flora compared to healthy children. The C. histolyticum group is recognized as a toxin producer, potentially contributing to gut dysfunction and systemic effects. GI problems were significantly more frequent in ASD patients than in controls (*P* < .05), with diarrhea being the most common GI symptom. A significant link was observed between the levels of the C. histolyticum group and GI problems in ASD patients (*P* < .001).
[91]	ASD	Case–Control	30 children (10 ASD, 10 NT, 10 PDD-NOS)	↑ Relative Abundance in ASD Group: *Clostridium, Caloramator, Sarcina,* Sutterellaceae*,* Enterobacteriaceae Higher levels of Bacteroidetes*, Alistipes, and Akkermansia* (vs. HC) ↓ Relative abundance in ASD Group: *Bifidobacterium spp.* Eubacteriaceae (except E. *siraeum*) ↑ Diversity seen in the ASD group according to Shannon and Chao indices	This study also showed that almost all identified Sutterellaceae (*Parasutterella* genus) and Enterobacteriaceae (e.g., *Proteus*, *Shigella*) were at higher levels in fecal samples of SAD children compared to the other controls. There was an increased Firmicutes/Bacteroidetes ratio in ASD children, primarily due to a reduction in Bacteroidetes
[92]	ASD	Case–Control with siblings	29 children	↑ Relative Abundance: *Desulfovibrio, Lactobacillus* (genera) in the ASD group ↓ Bacteriodetes/Firmicutes ratio in the ASD group	Similar to Strati et al. the Bacteroidetes/firmicutes ratio is decreased due to decreased Bacteroidetes and increased *firmicutes*.
[93]	ASD	Observational	12 total (6 ASD, 6 NT)	↑ *Faecalibacterium* genus	Differentially expressed genes in the ASD cohort are involved in IFN-gamma and Type I IFN signaling pathways, which may be associated with systemic immunity dysregulation and chronic inflammation
[94]	ASD	Case–Control	80 children (40 ASD, 40 HC)	↑ Relative Abundance: *Collinsella, Corynebacterium, Dorea, Lactobacillus* (genera) ↓ Relative Abundance: Bacteriodetes (phylum); *Veillonella, Dialister, Alistipes* (genera) ↑ Firmicutes/Bacteriodetes ratio (phylum)	Opposite phylum-ratio trends compared to other studies; suggests subgroup variance. ASD children had a significantly altered gut microbiota, particularly a higher Firmicutes/Bacteroidetes ratio. Multiple genera associated with SCFA production or inflammation were altered.
[95]	ASD	Case–Control	25 children (11 ASD, 14 HC)	↑ Relative Abundance Bacteroidetes, Proteobacteria ↑ Ratio of Bacteroidetes to Firmicutes ↓ Relative Abundance Actinobacteria, Actinocycetaceae*,* Bifidobacteriaceae*,* Gemellaceae *and* Streptococcaceae	A strong increase of Bacteroidetes and Proteobacteria and a decrease of Actinobacteria was observed in these patients. Reports an unbalance of gut microbiome structure with a shift in colonization by gut beneficial bacterial species in ASD patients in early childhood.
[96]	ASD	Case–Control	41 children (35 ASD, 6 HC)	Relative Abundance At the phylum level: ↑ Bacteriodetes/Firmicutes ratio in ASD children Genus level: ↑ *Sutterella, Odoribacter, Butyricimonas* ↓ *Veillonella, Streptococcus*	Butyrate/lactate producers reduced in ASD; microbial biomarkers are possible. This study adds to clinical evidence that altered gut microbiota could be linked to the development of ASD. With clinically significant results, they hypothesize that microbe-based disease analysis could predict novel connections between ASD and other diseases.
[97]	ASD	Shotgun Metagenomic	74 children (43 ASD, 31 HC)	↑ Simpson Diversity Index for Microbial Epitopes in ASD children compared to typically developing children	The inverse Simpson diversity index was significantly higher for the microbial epitopes (ME) from ASD children compared to typically developing (TD) children. Specific MEs are differentially represented: Increased MEs in ASD: Five MEs were significantly more abundant in the ASD children than TD children. Four of these were similar to peptides from human proteins GJA1, MYH7, PAX3, and EYA1. One ME from Listeria monocytogenes was also significantly increased in ASD children
[98]	ASD	Cross-sectional observational	136 children (78 ASD, 58 HC)	↑ Relative Abundance Bacteroidetes*, Parabacteroides, Sutterella, Lachnospira, Bacillus, Bilophila, Lactococcus, Lachnobacterium, Oscillospira* ↑ Ratio of Bacteroidetes to Firmicutes ↑ *β* Diversity	Significantly different beta diversity, with more diversity of ASD microbiota than the control group. This diversity shows a significant increase in the nine genera: *Bacteroides, Parabacteroides, Sutterella, Lachnospira, Bacillus, Bilophila, Lactococcus, Lachnobacterium, and Oscillospira* (*P* < .01)
[99]	ASD	Case–Control	60 total (30 ASD, 30 NT)	↑ *Clostridium boltaea, Clostridium histolyticum, and Clostridium difficile* in ASD	Increased relative abundance of three *Clostridium sp.,* associated with gastrointestinal symptoms in ASD children.
[100]	ASD	Comparative	247 total (99 ASD, 51 paired NT siblings, 97 unrelated NT)	*Romboutsia timonensis* was the only taxon associated with ASD	Only 1 taxon associated with ASD. ASD traits such as restricted eating habits are associated with less-diverse diets, which in turn are associated with lower alpha microbiome diversity.
[101]	ASD	Case–Control	86 total (45 ASD, 41 NT)	↓ Relative Abundance *Bacteroides,* Lachnospiraceae	Decreased relative abundance of Lachnospiraceae suggests correlation with pro-inflammatory cytokines IFN-gamma and IL-6. ASD group found to have significantly higher levels of IL-2, IL-4, IL-5, IL-6, IL-10, TNF-alpha, TNF-beta, INF-gamma.
[102]	ASD	Case–Control (with constipation subgroup)	80 children (40 C-ASD, 40 TD)	↑ Relative Abundance Ruminococcaceae_UCG_002, Erysipelotrichaceae_UCG_003, *Phascolarctobacterium*, *Megamonas*, *Ruminiclostridium*_5, *Parabacteroides*, *Prevotella*_2, *Fusobacterium*, *Prevotella*_9 ↓ *α*-diversity	SCFA levels: The C-ASD group had elevated levels of propionate in their feces compared to the TD group. Correlations: A negative correlation was observed between the abundance of Lactobacillus and fecal propionate levels, suggesting that lower Lactobacillus may be associated with higher propionate concentrations. Higher fecal propionate levels were positively correlated with increased severity of ASD symptoms.
[103]	ASD	RCT (Probiotic Study)	28 (16 ASD, 12 NT)	↑ Relative Abundance Actinobacteriota, Proteobacteria ↓ Relative Abundance Firmicutes	Children with ASD had an altered composition of gut microbiota in comparison with neurotypical children. After supplementation with a probiotic, ASD children's gut microbiome shifted to a more representative neurotypical gut microbiome. No significant *P*-value; however, using the percentile parameter, results showed a 12.4% improvement of autism symptoms in motor manifestations, visual reactions, fear and nervousness, nonverbal communication, and activity level in comparison to 6.6% improvement with placebo.
[104]	ASD	Comparative	8 total (4 ASD, 4 NT siblings)	↑ phyla Firmicutes and Proteobacteria in ASD ↑ genus Lactobacillaceae, *Bacteroides* ↓ relative abundance of *Bifidobacterium and*, *Prevotella* genus	Abundance of Firmicutes and Proteobacteria, increased diversity, richness, and biomass richness in ASD children
[105]	ASD	Phase I: Observational, Comparative, Phase II: Interventional Pilot	Phase I: 98 total (53 ASD, 45 NT) Phase II: 72 recruited, 53 completed	↑ *Prevotella sp., Dialister invisus,* and *Bacteroides sp.* in ASD children.	Increased specific microbiota in ASD children.
[106]	ASD	ML re-analysis of 3 16S datasets (discovery: sibling-matched case–control; 2 external validations)	117 total (60 ASD, 57 NT siblings)	↑ Prevotellaceae, *Parabacteroides* (Bacteroidota) ↑ multiple Lachnospiraceae/Clostridiaceae ASVs; two ↑ Enterobacteriaceae ASVs (Firmicutes & Proteobacteria) ↓ *Bifidobacterium*, *Collinsella* (Actinobacteria) ↓ *Butyricicoccus*, *Eubacterium eligens*, *Murdochiella*; one Enterobacteriaceae ASV ↑ in controls	REFS selected 26 discriminatory ASVs → average AUC = 0.816; best classifier (MLP) AUC = 0.90 External validation (223 samples) retained accuracy (mean AUC ≈ 0.75; best = 0.84) Signature suggests combined depletion of SCFA producers and enrichment of opportunistic taxa in ASD
[107]	Schizophrenia	Shotgun Metagenomic	16 SZ, 16 HC	↑ Relative Abundance *Ascomycota*, *Candida*, *Eubacterium* ↓ Relative Abundance *Neisseria*, *Haemophilus*, *Capnocytophaga*	*Ascomycota* were more abundant in the SZ cohort than control. The SZ cohort also had an increase in lactic acid bacteria, *Candida*, *Eubacterium* and a decrease in *Neisseria, Haemophilus,* and *Capnocytophaga*.
[108]	Schizophrenia	Phage-focused metagenomics	41 SZ, 33 HC	↑ Relative Abundance *Lactobacillus* phage phiadh	The bacteriophage composition in the oropharynx of individuals with schizophrenia differs from controls. *Lactobacillus phage phiadh* was significantly different in individuals with schizophrenia compared to controls (*P* < .00037, q < 0.03) Levels of *Lactobacillus phage phiadh* were higher in individuals with schizophrenia. Within the SZ group, *Lactobacillus phage phiadh* levels correlated with immunological disorders and valproate administration.
[109]	Schizophrenia	Cross-sectional	64 SZ, 53 HC	↑ Predominance in the SZ cohort Bacteriodetes, Firmicutes, Proteobacteria, Actinobacteria, fusobacteria ↑ Relative Abundance in the SZ cohort Proteobacteria	The abundance of Proteobacteria was higher in schizophrenia than in healthy controls. The abundance of *Succinovibrio* at the genus level was higher in the SZ cohort.
[110]	Schizophrenia	Observational + Mouse Study	63 SZ, 69 HC	↑ Relative Abundance Veillonellaceae ↓ Relative Abundance Lachnospiraceae*,* Ruminococcaceae ↓ *α*-diversity	Identifies an overlap in the key microbes involved in schizophrenia and depression. Some important limitations in the field of studying schizophrenia patients are the ethics of sources, unmedicated SZ patients, as well as the impacts of antipsychotics on test data.
[111]	Schizophrenia	Observational	38 SZ, 38 HC	↑ Relative Abundance: *Veillonella* ↓ Relative Abundance: *Ruminococcus, Roseburia*	Correlations Between Gut Microbiome and Brain Metrics Alpha Diversity and GMV: Higher alpha diversity (a measure of microbial richness and evenness) in SZ patients was positively correlated with GMV in the bilateral insula and right postcentral gyrus. *Roseburia* and ReHo: The abundance of *Roseburia* was negatively correlated with ReHo in the right STC, left cuneus, and right MTC. This suggests that lower levels of *Roseburia* may contribute to functional brain abnormalities in SZ
[112]	Schizophrenia	Mendelian Randomization	69,369 SZ; 236,642 controls	↑ Relative Abundance: Phyla: Firmicutes, *Acinetobacter* Classes: Betaproteobacteria, Clostridia, Order: Clostridiales Family: Prevotellaceae Genus: *Alloprevotella, Hungatella, Subdoligranulum* Protective: Family: Rhodospirillaceae, Defluviitaleaceae, Veillonellaceae Genus: *Coprobacter*, *Gordonibacter*, *Desulfovibrio*	The study identified mutual causal relationships between specific gut microbiota and schizophrenia. These findings suggest that targeting the gut microbiome could offer new avenues for understanding and potentially treating schizophrenia.
[113]	MDD (Depression + Parasite burden)	Cross-sectional, community-based	63 (Mexico)	↑ Relative Abundance *Ascaris lumbricoides*	The study found that *Ascaris lumbricoides* infections disrupted gut microbiota networks, reducing their complexity and emergence. This disruption was more pronounced in adults, suggesting a potential link between parasitic infections, gut microbiota imbalances, and depression.
[114]	MDD	Cohort (Observational)	2,593 total (Rotterdam + HELIUS)	↑ Relative Abundance: *Eggerthella, Sellimonas, Lachnoclostridium, Hungatella* ↓ Relative Abundance: *Ruminococcacae, Coprococcus, Lachnospiraceae, Rimonococcu gauvreauii* group, *Eubacterium ventriosum, Subdoligranulum, Ruminococcaceae* ↓ *α*-diversity	The study identified associations between depressive symptoms and specific gut microbiota, notably bacteria belonging to the families Christensenellaceae*,* Lachnospiraceae*, and* Ruminococcaceae. This study also ties their findings of Lachnoclostridium and depressive states to previous research. Alpha diversity was negatively associated with depressive symptoms.
[115]	MDD	Cross-sectional with severity stratification	138 MDD (mild, moderate, severe); 155 HC	↑ Relative Abundance *Bacteroides* ↓ Relative Abundance *Eubacterium, Ruminocaccaceae* ↓ *α*-diversity	Gut microbial composition changed with MDD severity, showing more pronounced dysbiosis in moderate and severe groups. A biomarker panel of 37 species was created using random forest classification, achieving high diagnostic accuracy (AUC = 0.992–0.998) in distinguishing severity levels. Functional analysis revealed certain metabolic genes (e.g., K12373, K21572) correlated with microbial shifts. The results suggest that gut microbiota may serve as non-invasive biomarkers for assessing MDD severity and potential therapeutic targets.
[116]	MDD & GAD Ind	Cross-Sectional observational	70 total (60 MDD + Anxiety, 10 NT)	↓ *Clostridium leptum* in depression patients*,* Bacteroides in anxiety patients	Reduced prevalence of *C.leptum* in depression patients compared to healthy controls, and reduced levels of Bacteroides in anxiety patients compared to cohorts independent of depression (comparing MDD to MDD + GAD; vice versa) (Note source overlap of anhedonia with GAD, not evaluated in this study)
[117]	Anxiety & Depression (IBD-related)	Cross-sectional	240 (UC + MDD + HC)	↑ Relative Abundance Lactobacillales, Sellimonas, *Streptococcus*, *Enterococcus*, Bacilli ↓ Relative Abundance *Prevotella*, *Lachnospira*	Patients with UC and depression/anxiety had distinct changes in their gut microbiota composition compared to those without these conditions. Composition characterized by a reduction in overall diversity and specific shifts in the abundance of certain bacterial groups.
[118]	GAD & MDD	Cross-sectional, case–control	10 HC, 21 GAD, 23 MDD	At the Genus Level: MDD vs. HC: MDD patients had significantly lower levels of *Fusicatenibacter* and *Sutterella*. GAD vs. HC: GAD patients showed significantly lower abundances of *Fusicatenibacter* and Christensenellaceae*_R7*_group GAD vs. MDD: Higher abundance of *Sutterella* and lower abundance of *Faecalibacterium* in GAD compared to MDD.	Microbial diversity and richness: MDD vs. HC: Statistical analysis suggested there was not sufficient evidence to say there was a difference in richness and diversity between the two groups. GAD vs. HC: GAD patients had increased richness and diversity that was statistically significant, but the OTU abundance was not significant. GAD vs. MDD: Microbiota showed similarities that did not have a statistically significant difference, suggesting the microbes in these disorders may be similar in composition.
[119]	GAD & MDD	Case-Control	69 total (23 anxiety + depression, 46 NT)	*Prevotella* is the dominant genus in anxiety + depression group, *Faecalibacterium* genus in the control group. ↓ relative abundance of *Gemmiger*, *Ruminococcus*, and *Veillonella*	Dominant species varied between control and experimental groups, and a lower relative abundance of *Gemmiger, Ruminococcus,* and *Veillonella* may be linked with lower SCFA production.
[120]	GAD & MDD	Mendelian Randomization	GWAS (7,738 Dutch; 18,340 MiBioGen; 13,559 depression; 1,092 anxiety cases)	Depression: ↑ Abundance - increased risk of depression *Bilophila* genus ↓ Abundance - decreased risk of depression *Escherichia Shigella* Anxiety: ↑ Abundance - increased risk of anxiety *Clostridium innocuum* (potentially through neuroactive substances such as GABA, acetylcholine). ↓ Abundance - decreased risk of anxiety *Pseudoflavonifractor capillosus* and *Ruminococcus* (potentially through production of SCFA)	The study provided strong evidence of a strong relationship between specific gut microbiota taxa and depression/anxiety disorders. Certain bacterial taxa could serve as potential biomarkers or therapeutic targets for these conditions.
[121]	GAD	Cross-sectional	82 GAD, 97 HC	↑ Relative Abundance *Bacteroides* *↑ Relative Abundance positively correlated with symptom severity*	This study highlights significant differences in gut microbiota, diet, and metabolic profiles between GAD patients and healthy controls. The Bacteroides/fiber ratio, stool acetate levels, and GI symptoms emerged as strong predictors of anxiety disorder status.
[84]	Early-life Anxiety	Observational	77 toddlers (18–27 months)	↑ Phylogenetic diversity in children with greater surgency/extraversion	Greater Surgency/Extraversion was associated with greater phylogenetic diversity among both boys and girls. Additional sex-specific associations were observed in relation to phylogenetic diversity, the Shannon Diversity Index (SDI), beta diversity, and abundances of specific bacteria. Bidirectional brain-gut relationships may exert measurable effects in humans in early life.
[122]	Social anxiety disorder	Case–Control Study	31 SAD, 18 HC	↑ Relative Abundance *Anaeromassilibacillus* and *Gordonibacter*	The research identified distinct differences in the gut microbiome's composition and function between those with SAD and healthy individuals. Notably, the metabolic pathway associated with aspartate degradation was more active in the gut microbiota of SAD patients. These findings suggest that alterations in gut microbiota may play a role in the development or manifestation of social anxiety disorder.

Note. Arrows indicate the direction of change in the patient group relative to the comparison group (↑ = higher/diversity increased; ↓ = lower/diversity reduced). n refers to the number of human participants analyzed per study (sub-groups shown in parentheses). ASD: Autism spectrum disorder; C-ASD: Constipation-associated ASD subgroup; GAD: Generalized anxiety disorder; MDD: Major depressive disorder; SAD: Social anxiety disorder; SZ: Schizophrenia; UC: Ulcerative colitis; HC: Healthy controls; NT: Neurotypical controls; IBD :Inflammatory bowel disease; TD: Typically developing children; GWAS: Genome-wide association study; MR: Mendelian randomization study; RCT: Randomized controlled trial; SCFA: Short-chain fatty acid; OUT: Operational taxonomic unit; SDI: Shannon Diversity Index; GMV: Grey-matter volume; ReHo: Regional homogeneity; STC / MTC: Superior / Middle temporal cortex; Mes: Microbial epitopes; GI: Gastrointestinal; PDD-NOS: Pervasive developmental disorder–not otherwise specified; ML: Machine Learning; ASV: Amplicon sequence variant; RFS: Recursive feature selection; MLP: Multilayer perceptron; Extra Trees: Extremely randomized trees; AUC: Area under the receiver; ROC: Receiver operating characteristic curve.

### 3.1. Shared and divergent gut microbiota dysbiosis patterns across neuropsychiatric disorders

Alterations in gut microbiota alpha diversity show disorder-specific patterns. While depression consistently exhibits reduced gut microbial richness and evenness,^114^^,^^115^ findings in SZ are mixed, with some cohorts reporting decreased alpha diversity,^110^ and others noting preserved alpha diversity but compositional alterations.^111^ In contrast, GAD patients exhibit significantly increased richness and diversity compared to healthy controls.^118^^,^^121^ Similarly, certain ASD studies report elevated phylogenetic or Simpson diversity indices.^91^^,^^97^^,^^98^

Multiple neuropsychiatric disorders exhibit reduced populations of beneficial bacteria that produce SCFA. Ruminococcaceae family members are consistently reduced in MDD, with specific decreases in *Ruminococcus* gauvreauii group and other Ruminococcaceae taxa.^114^^,^^115^ SZ shows similar patterns with decreased Ruminococcaceae and Lachnospiraceae families.^110^ ASD cohorts show reduced Lachnospiraceae in some studies.^101^ Specific SCFA-producing genera, including *Gemmiger*, *Ruminococcus*, and *Veillonella* are depleted in anxiety-depression comorbid presentations.^119^

Patterns of *Bacteroides* abundance vary across conditions. GAD patients demonstrate elevated *Bacteroides* abundance that positively correlates with symptom severity measures.^121^ Similar increases have been reported in MDD,^115^ and in certain ASD cohorts.^98^^,^^104^ In contrast, another study found significantly reduced *Bacteroides* abundance in anxiety patients, independent of comorbid depression.^116^

Several bacterial taxa display distinct trends across neuropsychiatric conditions. *Sutterella* is consistently increased in ASD cohorts^96^^,^^98^ and shows higher abundance in GAD compared to MDD, but appears reduced in MDD patients specifically.^118^ *Faecalibacterium* exhibits more complex patterns, with some ASD studies reporting elevated levels,^93^ while reductions have been observed in GAD compared to MDD^118^ and in anxiety–depression groups relative to healthy controls.^119^

### 3.2. Condition-specific microbial signatures

ASD is characterized by prominent phylum-level alterations, with most studies documenting increased Bacteroidetes relative to Firmicutes,^95^^,^^96^^,^^98^^,^^123^ though patterns are cohort-dependent, with contradictory findings across other studies.^91^^,^^92^^,^^94^ Proteobacteria enrichment is also commonly observed.^95^^,^^103^^,^^104^ *Clostridium* species, particularly *Clostridium histolyticum*, *Clostridium difficile*, and related toxin-producing strains, show consistent elevations, especially in ASD children presenting with GI symptoms.^90^^,^^91^^,^^99^ While restrictive diets have been proposed as a driver of microbial changes,^100^ other evidence supports a causal role. Machine learning approaches have successfully identified discriminatory bacterial signatures with high diagnostic accuracy for ASD classification.^106^

*SZ* is marked by reductions in both alpha and beta diversity across multiple cohorts.^107^^,^^110^^,^^111^^,^^124^ Lactic acid-producing bacteria, including *Bifidobacterium* and *Lactobacillus* show increased abundance in oropharyngeal microbiomes,^107^ while Proteobacteria levels are elevated.^109^ Unique microbiota features include enrichment of Veillonellaceae and Ascomycota, with specific increases in *Candida* and *Eubacterium.*^107^^,^^110^ Oropharyngeal microbiota alterations include elevated *Lactobacillus* phage phiadh levels that correlate with immune abnormalities and medication use.^108^ Functional preclinical studies have shown that microbiota transplantation from SZ patients can induce behavioral abnormalities and altered brain gene expression in germ-free mice.^125^^,^^126^

MDD is distinguished by specific taxonomic enrichments including *Eggerthella*, *Sellimonas*, *Lachnoclostridium*, and *Hungatella*, with concurrent decreases in beneficial Ruminococcaceae, *Coprococcus*, Lachnospiraceae, and *Eubacterium ventriosum.*^114^^,^^115^ *Bilophila* genus abundance positively associates with depressive symptoms, while higher *Escherichia*-*Shigella* levels appear protective.^120^ Parasitic infections, particularly *Ascaris lumbricoides*, can disrupt gut microbiota network complexity and correlate with depressive symptomatology.^113^ Multi-omics analysis in irritable bowel disease-related depression reveals distinct bacterial compositions characterized by increased Lactobacillales, *Sellimonas*, *Streptococcus*, and *Enterococcus*, with decreased *Prevotella* and *Lachnospira.*^117^

Social anxiety disorder exhibits distinct microbiota compositions with increased *Anaeromassilibacillus* and *Gordonibacter* genera, alongside enhanced aspartate degradation pathways.^122^ GAD specifically shows elevated *Bacteroides* that correlate with symptom severity, decreased stool acetate levels, and altered *Bacteroides*/fiber ratios that predict disorder status.^121^ Inflammatory markers, including IL-1β, IL-6, IL-17, and TNF-*α* are consistently elevated in GAD patients.^127^ Genetic studies identify specific risk-associated taxa, with *Clostridium innocuum* linked to increased anxiety risk and *Pseudoflavonifractor capillosus* and *Ruminococcus* showing protective effects, potentially through SCFA production.^120^

### 3.3. Functional and mechanistic convergence

Despite taxonomic differences, microbial alterations across neuropsychiatric disorders converge on shared functional pathways. Inflammatory cytokine elevations, including IL-6, TNF-*α*, IL-1β, IL-17, and IFN-*γ* are documented across ASD, SZ, MDD and GAD.^101^^,^^127^^,^^128^ Complement pathway disruptions and synaptic pruning mechanisms provide mechanistic links between gut dysbiosis and neuroinflammation.^87^^,^^129^

## 4. Gut microbiota dysbiosis in skin diseases

The human skin functions as both a protective barrier and an active immune organ while maintaining physiological homeostasis. Given the frequent co-occurrence of neuropsychiatric and dermatological disorders, researchers have investigated whether skin conditions exhibit similar gut microbiota dysbiosis patterns. Clinical studies across atopic dermatitis, chronic spontaneous urticaria (CSU), psoriasis, rosacea, seborrheic dermatitis, and eczema reveal overlapping yet distinct microbial alterations that influence systemic nutrient availability and immune regulation.

**Figure 2.**
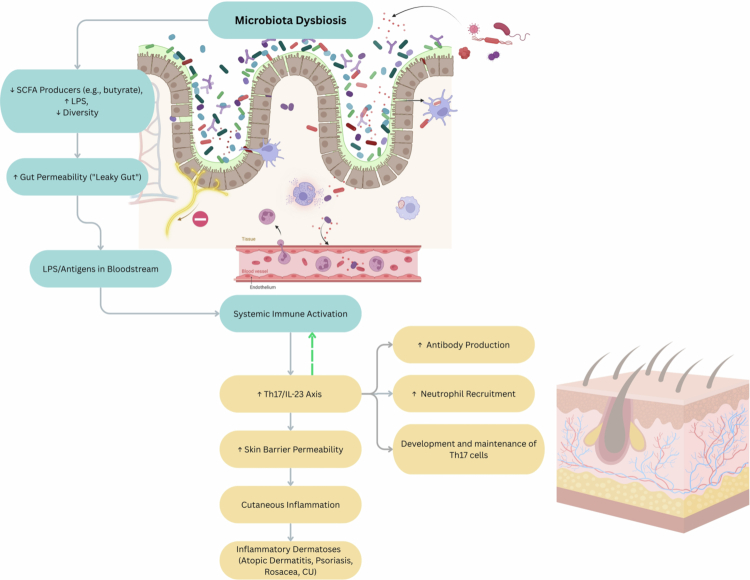
Microbiota dysbiosis and cutaneous inflammatory dermatoses via gut–skin axis disruption. This schematic illustrates how microbiota dysbiosis contributes to the development of inflammatory skin conditions via gut–skin axis signaling. Dysbiosis is characterized by reduced SCFA producers, increased lipopolysaccharide (LPS) levels, and decreased microbial diversity. This imbalance leads to impaired intestinal barrier function and translocation of microbial antigens into the bloodstream. This activates systemic immune pathways. Within the cutaneous tissue, the T-helper 17/interleukin-23 (Th17/IL-23) axis further promotes systemic immune responses, while also facilitating neutrophil recruitment, antibody production, and the continued maintenance of Th17 cells. These processes disrupt skin barrier integrity, foster cutaneous inflammation, and contribute to inflammatory dermatoses, including atopic dermatitis, chronic urticaria (CU), psoriasis, and rosacea. The green dashed arrow denotes the positive feedback of the Th17/IL-23 axis to the immune processes systemically. Created in BioRender. Hawkins, B. (2025)https://BioRender.com/adla0kf. Final figure graphics made in Canva.

As summarized in Table 2, clinical studies of the aforementioned dermatological conditions reported gut microbiota alterations, which influence systemic nutrient availability, particularly SCFA.^130–133^ Among SCFA, butyrate is notable for modulating skin immune responses and strengthening the epidermal barrier.^134–136^ SCFA levels are strongly influenced by the relative abundance of Firmicutes and Bacteroidetes in the gut,^133^^,^^137^ highlighting how shifts in microbial composition can affect skin health. Figure 2 illustrates potential gut–skin axis mechanisms linking gut dysbiosis to dermatological outcomes. 

**Table 2.** Clinical studies of gut microbiota dysbiosis in skin diseases.

Reference	Skin disease	Type of study	Sample size	Microbiota changes identified	Key findings
[138]	Atopic Dermatitis	Prospective birth cohort (Netherlands)	957 infants (post-exclusion)	↑ Prevalence in infants who developed eczema: Bifidobacteria (98.7%), *Escherichia coli* (88.6%), Bacteroides fragilis group (81.6%) ↓ Prevalence: *Lactobacilli* (32.2%), *Clostridium difficile* (25.1%)	Colonization with C. *difficile* was linked to a higher risk of eczema. Increased E. coli correlated with higher eczema risk, regardless of C. *difficile* levels.
[139]	Atopic Dermatitis	Comparative observational study	19 AD children, 18 healthy controls	↑ Relative abundance: *Faecalibacterium, Oscillospira, Bacteroides, Parabacteroides, Sutterella* ↓ Relative abundance: *Bifidobacterium, Blautia, Coprococcus, Eubacterium,* Propionibacterium*, Actinomyces,* Eggerthella*,* Enterococcus*,* Staphylococcus*,* Corynebacterium*, Bulleidia*	AD-associated dysbiosis was linked with impaired immune regulation and reduced anti-inflammatory metabolites.
[140]	Atopic Dermatitis	Cross-sectional study	23 AD (5–11-year-olds), 58 controls	↑ Relative abundance: Bifidobacteria ↓ Relative abundance: Lactobacilli	Gut microbiota differences were shown in children with AD compared to school-aged children without allergic disease, regardless of socioeconomic status.
[141]	Atopic Dermatitis	Prospective controlled observational trial	Patients: eczema (*n* = 19), hives (*n* = 9), rhinitis (*n* = 11)	↑ Relative abundance: Bacteroidales (order), Bacteroidia (class), Bacteroidetes (phylum), *Romboutsia*, *Sutterella*	Significant gut microbiota differences were observed across allergic disease subgroups, especially in eczema.
[142]	Atopic Dermatitis	Two-sample Mendelian randomization	GWAS data: gut (*n* = 18,340), AD (*n* = 218,467)	↑ Genetic association with increased AD risk: Clostridiaceae*_1,* Bacteroidaceae (families); *Bacteroides, Anaerotruncus,* Lachnospiraceae UCG001, Eubacterium hallii group, and an unknown genus ↓ Genetic association with reduced AD risk (protective taxa): Tenericutes (phylum); Mollicutes*,* Clostridia*,* Bacilli (classes); Bifidobacteriales (order); Bifidobacteriaceae (family); *Bifidobacterium*, *Christensenellaceae* R7 group, *Anaerostipes* (genera)	Provides genetic evidence of gut microbiota's role in AD pathogenesis; suggests microbiome-targeted therapy potential.
[143]	Seborrheic Dermatitis	Two-sample Mendelian randomization and bidirectional MR	GWAS data: SD (*n* = 339,277), gut microbiota (*n* = 18,340 across 24 cohorts)	↑ Risk-associated (genetic): Tenericutes*,* Firmicutes*,* Mollicutes*, Senegalimassilia, Victivallis* ↓ Protective (genetic): *Butyrivibrio, Eubacterium eligens group, Howardella,* Lachnospiraceae NC2004 group*, Ruminiclostridium 5*	Five bacterial taxa were associated with increased SD risk; others showed a protective effect. The study suggests the gut microbiota may play a significant role in SD pathogenesis.
[144]	Chronic Urticaria	Case-control study	25 patients with symptomatic dermographism, 25 controls	↓ Relative abundance: *Subdoligranulum*, *Ruminococcus bromii*	Highlights microbiota variation across CSU subtypes.
[145]	Chronic Urticaria	Cross-sectional study	30 CSU patients, 40 controls	↑ Relative abundance: Lactobacillaceae*,* Turicibacteraceae*,* Peptostreptococcaceae*, Lachnobacterium, Turicibacter, Lactobacillus* ↓ Relative abundance: Veillonellaceae*, Phascolarctobacterium*	Gut microbiota differences in CSU patients may offer future biomarker potential.
[146]	Chronic Urticaria	Case-control study	22 CSU patients, 23 controls	↑ Relative abundance: *Lactobacillaceae*, *Lactobacillus* ↓ Relative abundance: *Lachnospiraceae* (including *Lachnospira, Roseburia, Ruminococcus, Coprococcus, Eubacterium eligens*), *Barnesiellaceae*, *Butyricicoccaceae*, *Carnobacteriaceae*	Lachnospiraceae, as SCFA producers, may be protective against CSU; significant microbiota differences observed.
[147]	Chronic Urticaria	Prospective cohort study	26 CSU patients, 26 controls (stool), and additional plasma samples for sequencing	↓ *α*-diversity ↓ SCFA producers: Rikenellaceae (family), *Alistipes* (genus), *Roseburia hominis* (species)	CSU patients had significantly fewer SCFA-producing gut microbes.
[148]	Psoriasis	Observational study	32 psoriasis patients, 64 controls	↑ Relative Abundance: Firmicutes (phylum), Ruminococcaceae and Lachnospiraceae (families), *Ruminococcus, Megasphaera, Dialister,* and *Dorea* (genera) ↓ Relative Abundance: Bacteroidetes (phylum), Bacteriodaceae and Prevotellaceae (families), *Akkermansia* (genus), *Prevotellae* and *Paraprevotella* (genera)	Altered gut microbiota linked to changes in metabolic enzyme activity.
[149]	Psoriasis	Prospective observational study	52 patients, > 300 healthy controls	↑ Overall abundance: *Akkermansia*, *Faecalibacterium* ↓ Overall abundance: *Bacteroides* Predominance amongst: Enterotype 1: ↑ *Bacteroides* Enterotype 2: ↑ *Prevotella* ↓ *Bacteroides* to *Faecalibacterium* ratio Enterotype 3: ↑ *Ruminococcus*	Microbiota significantly differed between psoriatic and healthy individuals. Principal Component Analysis (PCA) is used to analyze samples against controls. Bacterial DNA translocation (BT) blood samples were used to compare between enterotypes and controls.
[150]	Psoriasis	Observational multicenter study	52 participants (*n* = 24 with psoriasis)	↑ Relative abundance: Firmicutes (phylum), Actinobacteria (phylum), *Ruminococcus gnavus, Dorea formicigenerans, Collinsella aerofaciens* ↓ Relative abundance: Bacteroidetes (phylum) ↓ *Prevotella copri* and *Parabacteroides distasonis*	Significant differences in *β*-diversity, despite similar *α*-diversity.
[151]	Psoriasis	Cross-sectional study	32 psoriasis patients, 15 healthy controls, 17 healthy partners of PS patients	↑ Relative abundance: Bacteroidetes (phylum) Bacteroides uniformis ↓ Relative abundance: Firmicutes (phylum), *Roseburia*, *Eubacterium* (genera), *Roseburia hominis* (species)	Strong association between intestinal flora and psoriasis.
[152]	Rosacea	Prospective study	113 rosacea patients, 60 controls	↑ Prevalence: Small intestinal bacterial overgrowth (SIBO) in rosacea patients compared to controls	Eradication of SIBO led to significant lesion clearance.
[153]	Rosacea	Cross-sectional study	12 rosacea patients, 251 controls (after exclusion)	↑ Relative abundance: *Acidaminococcus*, *Megasphaera*, Lactobacillales (unknown genus) ↓ Relative abundance: *Slackia*, *Coprobacillus*, *Citrobacter*, *Desulfovibrio*, Peptococcaceae (unknown genus), *Methanobrevibacter*	Significant *β*-diversity differences despite similar *α*-diversity.
[154]	Rosacea	Two-sample Mendelian randomization	GWAS data: gut (*n* = 18,340), rosacea (*n* = 69,374 cases and 236,642 controls)	↓ Genetic association with rosacea risk: Actinobacteria (phylum), *Butyrivibrio* (genus) Nominal causal associations were also observed for 14 additional taxa (not FDR-significant)	Suggests a nominal causal role of gut microbiota in rosacea.
[155]	IgE-associated Eczema	Clinical and RCT	10 eczema patients, 10 non-allergic infants	↓ Relative abundance: Ruminococcaceae (family) in infants at 1 week of age who later developed IgE-associated eczema	Early life microbiota differences may predict eczema development.

AD: Atopic dermatitis; BT: Bacterial translocation; SD: Seborrheic dermatitis; CSU: Chronic spontaneous urticaria; FDR: False discovery rate; GWAS: Genome-wide association studies; MR: Mendelian randomization; PCA: Principal component analysis; PS: Psoriasis; RCT: Randomized controlled trial; SCFA: Short chain fatty acids; SIBO: Small intestinal bacterial overgrowth.

**Table 3.** Clinical applications of probiotics for gut microbiota modulation in skin diseases.

Reference	Skin condition	Study type	Participants	Intervention	Important findings
[156]	Atopic Dermatitis	RCT	50 children, ages 4−17, on a high-quality Mediterranean diet (KIDMED score qualifier)	All 50 patients received topical steroids, moisturizers, and an oral antihistamine. Patients in the probiotic group received a pill to be taken daily with three probiotic strains: *Bifidobacterium lactis* CECT 8145, *B. longum* CECT 734, and *L. casei* CECT 910.	96% of patients receiving the oral probiotic exhibited a decrease in SCORAD score.
[157]	Atopic Dermatitis	Randomized, double-blind, placebo-controlled trial.	43 children, ages 4−17, diagnosed with moderate AD (21 in the placebo group, 22 in the probiotic group)	Patients received a daily probiotic pill containing *Bifidobacterium animalis lactis* CECT 8145, *Bifidobacterium longum* CECT 7347, and *Lacticaseibacillus casei* CECT 9104, or a placebo for 12 weeks. SCORAD was measured every 4 weeks.	Lower SCORAD scores were associated with a decrease in *Faecalibacterium* and an increase in *Bifidobacterium* within the probiotic group.
[158]	Atopic Dermatitis	RCT (double-blind, placebo-controlled)	91 (6 months - 3-year-old) patients with atopic dermatitis; 46 in intervention, 45 controls	*Lacticaseibacillus rhamnosus* GG was given once daily for 12 weeks; the control group was given a placebo pill once daily. The efficacy of the treatment was assessed 1 month (4 weeks) after treatment completion.	LGG daily supplementation was effective in reducing the SCORAD index, topical steroid use, and improving the quality of life in pediatric patients. Meta-analysis also showed that longer treatment duration (>8 weeks) provided the most significant benefit in children with AD.
[159]	Chronic Spontaneous Urticaria	Randomized placebo-controlled trial	206 children with confirmed diagnoses of CSU were assigned to either the probiotic group (104 children) and placebo group (102 children)	Participants in the probiotic group received Yimingja, a combination probiotic product of 6 organisms (5 *Lactobacillus* strains and 1 *Bifidobacterium* strain) orally twice daily for 4 weeks	Probiotic supplementation reduced wheal size, and attack frequency from the first week of treatment until the end of the 4th week was improved
[160]	Chronic Spontaneous Urticaria	Blinded RCT	42 patients with CSU; 21 patients in the control group receiving only antihistamines, and 21 in the intervention group receiving antihistamine + LactoCare oral synbiotic (prebiotic + probiotic)	Patients in the intervention group received LactoCare oral probiotic capsules twice a day for 8 weeks. All patients received two of the following three oral antihistamines twice a day: Cetirizine 10 mg, Desloratadine 5 mg, and Fexofenadine 180 mg	Using probiotics as adjunctive therapy is effective in reducing the severity of itching and frequency of urticaria episodes. There was no statistically significant difference in efficacy between the patients receiving antihistamines alone vs the antihistamine + synbiotic group based on UAS7 scores.
[161]	Chronic Spontaneous Urticaria	Randomized Clinical Trial	38 patients with CSU unresponsive to antihistamines; 18 patients in the control group and 20 patients in the intervention group	Patients in the intervention group received an antihistamine (Cetirizine) and probiotics (Femilact capsule); the control group received an antihistamine (Cetirizine) and a placebo. Both groups received an antihistamine and a probiotic or a placebo twice a day for 8 weeks.	The intervention group showed a significant improvement in CSU symptoms and a reduction in UAS7 scores. There was no significant difference in Quality-of-Life scores between the control and intervention groups.
[162]	Psoriasis	RCT (double-blind, placebo-controlled)	26 psoriasis patients (Intervention: 13; Control: 13)	*Bifidobacterium infantis* 35624 administered orally for 6−8 weeks	Significant reduction in plasma CRP levels across all patient groups. TNF-*α* levels decreased significantly in psoriasis patients. Demonstrated systemic anti-inflammatory effects beyond the gut.
[163]	Psoriasis	RCT (double-blind, placebo-controlled)	90 psoriasis patients, aged 18−70 years old (Intervention: 46; Control: 44)	Adjuvant treatment with topical corticosteroid, betamethasone, in combination with calcipotriol. Participants in the intervention group received a probiotic mixture of *Bifidobacterium, B. Lactis* and *L. rhamnosus.*	The probiotic blend showed a reduction in the severity of psoriasis when administered with a topical corticosteroid. A higher proportion of patients classified as clear or almost clear in the PGA index with a lower need for the prescription of betamethasone steroids. The disappearance of *Micromonospora* and *Rhodococcus* and an increase of the positive genera *Collinsella* and *Lactobacillus* in the group receiving probiotics.
[164]	Psoriasis	RCT (double-blind, placebo-controlled)	35 psoriasis patients > 18 years old (Intervention: 18; Control: 17)	*Lactobacillus rhamnosus* Lr-G14 (5 × 10⁹ CFU/g, one capsule daily) for 60 days alongside standard psoriasis medications	Significant improvements in plaque numbers and PASI, BSA, and DLQI scores indicate a reduction in disease severity. No significant changes in IL-17 and IL-23 levels between groups. Minimal adverse events.
[165]	Psoriasis	RCT (double-blind, placebo-controlled)	103 psoriasis patients in Brazil (Intervention: 50; Control: 53)	Standard-of-care plus *Lactobacillus rhamnosus* formula (6 × 10⁵ CFU/ml, 5 ml daily) for 6 months	No significant clinical benefits from probiotic treatment. Intervention group showed a non-significant PASI decrease (1.58 points, *P* = .105) The control group showed significant improvement (1.90 points, *P* = .019). Findings do not support gut microbiome modulation via *L. rhamnosus* for psoriasis treatment.
[166]	Psoriasis	Non-Randomized, Single Center Clinical Trial	63 psoriasis patients (Intervention: 42; Control: 21)	6 Bacillus strain probiotics and precision prebiotics	Overall improvement in psoriasis symptoms. Increased gut microbiota diversity and modulated healthy microbial population ratios.
[167]	Rosacea	Case Report	37-year-old with a two-year history of scalp rosacea	Treated with an 8-week course of doxycycline 40 mg once a day and probiotic therapy (*Bifidobacterium* breve BR03, *Lactobacillus salivarius* LS01 1 × 10^9 UFC/dose) twice a day.	Significant improvement of scalp rosacea with crusting and pustules completely disappeared
[168]	Rosacea	Randomized, placebo-controlled clinical trial	60 rosacea patients were randomly assigned to three groups: probiotic, placebo, or control. After 2 weeks of doxycycline treatment, participants underwent a 3-month intervention with either a placebo, probiotic, or no further treatment.	All participants initially received a 2-week doxycycline treatment of hydrochloride (50 mg per administration, twice daily). Participants in the probiotic group received a probiotic comprised of 9.70 Log10 CFU of *Lacticaseibacillus paracasei*, 9.70 Log10 CFU of *Lactiplantibacillus plantarum*, 9.70 Log10 CFU of *Lacticaseibacillus rhamnosus*, Probio-M9, 9.88 Log10 CFU of *Bifidobacterium (B.) animalis lactis* V9 and 9.88 Log10 CFU of *B. animalis lactis* Probio-M8.	The probiotic intervention had a symptom-relieving effect on rosacea with significantly lowered PGA scores, decreased TNF-(alpha) levels, and improved SC hydration, with a significant reduction in facial skin microbiota diversity while improving gut microbiota heterogeneity. No notable changes in the alpha diversity of the fecal microbiota.
[169]	Rosacea	Experimental, Randomized, Controlled, Double-blind Clinical Study	25 patients with erythematotelangiectatic and papulopustular rosacea were divided into 2 groups: the experimental group and the control group.	Both the experimental and control groups used the topical cream Adapalene 1 mg every other day on the face. Participants in the experimental group received the FQM Melora probiotic Probiac, two tablets once a day for one month. The probiotic was composed of a combination of *Lactobacillus acidophilus*, *Lactobacillus delbrueckii bulgaricus,* and *Bifidobacterium bifidum*. The control group used placebo tablets of oral probiotics.	Observational improvement in rosacea symptoms was observed but no evidence of clinical superiority of treatment when comparing the probiotic and placebo.
[170]	Eczema	Double-blind, Randomized, Placebo Controlled Trial	474 children; 159 received placebo, 157 received *L. rhamnosus,* 158 received *B. animalis* subsp lactis HN109	Probiotic supplements *L. rhamnosus* HN001 and *B. animalis lactis* HN019	Treatment with *L. rhamnosus* HN001 for the first 2 years of life showed a prevalence reduction of about half for any type of eczema and protection against having a SCORAD value. Neither probiotic showed a statistically significant effect against atopic sanitation.

BSA: Body surface area; CFU: Colony forming units; CRP: C-reactive protein; AD: Atopic dermatitis; CSU: Chronic spontaneous urticaria; DLQI: Dermatology life quality index; IL: Interleukin; KIDMED: Mediterranean diet quality index for children and adolescence; PASI: Psoriasis area and severity index; PGA: Physician's global assessment; RCT: Randomized controlled trial; SC: Stratum corneum; SCORAD: SCORing atopic dermatitis; TNF-α: Tumor necrosis factor-alpha; UAS7: Urticaria activity score over 7 days; UFC: Unità Formanti Colonie (Colony Forming Units in Italian).

### 4.1. Convergent and differing patterns of gut microbiota dysbiosis in dermatological conditions

The most consistent finding across dermatological conditions involves compromised populations of bacteria that produce beneficial SCFA. CSU exhibits marked reductions in Lachnospiraceae family members, including *Lachnospira*, *Roseburia*, *Ruminococcus*, *Coprococcus*, and *Eubacterium eligens,*^146^ alongside decreased *Roseburia hominis*, *Ruminococcus bromii*, and *Subdoligranulum* in multiple cohorts.^144^^,^^147^ Psoriasis studies show decreased *Roseburia* and *Eubacterium* genera across different populations.^151^ Seborrheic dermatitis genetic analyses identify protective associations with *Butyrivibrio*, *Eubacterium* eligens group, and Lachnospiraceae NC2004 group.^143^ Early-life eczema development correlates with reduced Ruminococcaceae family abundance in the first week of life.^155^

Reduced microbial diversity appears as a common feature in several skin conditions. CSU consistently demonstrates decreased alpha diversity across multiple studies,^143^ whereas other skin conditions may maintain overall diversity but exhibit distinct beta diversity profiles.^153^

Complex patterns emerge for lactic acid-producing bacteria across skin conditions. CU is associated with a consistent increase in *Lachnobacterium*, *Lactobacillus*, and *Turicibacter* abundance,^145^^,^^146^ whereas patients with rosacea show elevated Lactobacillales levels.^153^ Conversely, some atopic dermatitis cohorts exhibit decreased Lactobacilli,^140^ and early colonization patterns indicate that infants who later develop eczema also exhibit lower Lactobacilli prevalence.^138^

### 4.2. Disease-specific microbial patterns

Atopic dermatitis presents complex age-dependent patterns. Older children and adults typically show enrichment of the Bacteroidales members, including *Bacteroides*, *Parabacteroides*, and *Sutterella*, with relative depletion of beneficial SCFA-producing taxa *Bifidobacterium*, *Blautia*, *Coprococcus*, and *Eubacterium.*^139^^,^^141^ In contrast, some infant cohorts show increased *Bifidobacteria* and decreased Lactobacilli.^140^ Genetic studies support the protective effects of increased *Bifidobacterium*, Christensenellaceae, and *Anaerostipes* abundance.^142^ Furthermore, early colonization studies reveal that *Clostridium difficile* and *E. coli* correlate with increased eczema risk regardless of other bacterial levels.^138^

For *Seborrheic dermatitis*, limited research reveals genetic risk associations through Mendelian randomization approaches. Risk-associated taxa include Tenericutes, Firmicutes, Mollicutes, *Senegalimassilia*, and *Victivallis*, while potential protective associations involve *Butyrivibrio*, *Eubacterium* eligens group, *Howardella*, Lachnospiraceae NC2004 group, and *Ruminiclostridium* 5.^143^

CSU is characterized by consistent taxonomic shifts, including increased Lactobacillaceae, Turicibacteraceae, Peptostreptococcaceae, as well as specific genera such as *Lachnobacterium*, *Turicibacter*, and *Lactobacillus.*^145^ Concurrent decreases occur in Veillonellaceae, beneficial SCFA producers, and specific taxa, including *Phascolarctobacterium.*^145^^,^^146^ Additionally, one study reported reduced alpha diversity accompanied by a lower abundance of protective genera such as *Alistipes.*^147^

Psoriasis exhibits variable patterns across different populations and study methodologies. Some cohorts show increased Firmicutes with decreased Bacteroidetes, alongside depletion of Ruminococcaceae, Lachnospiraceae, and genera including *Ruminococcus*, *Megasphaera*, *Dialister*, and *Dorea.*^148^^,^^150^ Other populations show contradicting phylum-level patterns with increased Bacteroidetes and decreased Firmicutes.^151^ Despite these variations, consistent findings include altered *Akkermansia* and *Faecalibacterium* levels across cohorts^149^ and decreased *Roseburia* and *Eubacterium* genera.^151^ Bacterial DNA translocation studies reveal distinct enterotype patterns with varying *Bacteroides* to *Faecalibacterium* ratios.^149^

Rosacea is associated with increased prevalence of small intestinal bacterial overgrowth, with therapeutic SIBO eradication leading to significant lesion improvement.^152^ Taxonomic analyses reveal gender-specific patterns, with female patients showing increased *Acidaminococcus*, *Megasphaera*, and Lactobacillales of unknown genus, alongside decreased *Slackia*, *Coprobacillus*, *Citrobacter*, *Desulfovibrio*, and *Peptococcaceae.*^153^ Genetic analyses suggest protective effects of higher *Butyrivibrio* and Actinobacteria against rosacea, though additional taxa show nominal causal associations.^154^

### 4.3. Mechanistic convergence and clinical implications

The consistent depletion of SCFA-producing bacteria across multiple skin conditions suggests shared mechanisms involving compromised gut barrier integrity, reduced anti-inflammatory metabolite production, and altered immune tolerance. SCFA, particularly butyrate, simultaneously strengthen the epidermal barrier and promote immune tolerance (skin resident regulatory T-cells); commensals such as *Bacteroides fragilis* and certain *Clostridia* can induce Foxp3 + Tregs and tolerance.^134^^,^^135^^,^^171^ Dysregulation of commensal microbes and altered epithelial repair mechanisms that are mediated through pattern recognition receptors may predispose individuals to chronic inflammatory skin conditions.

Important distinctions exist between broad “eczema” classifications and specific “atopic dermatitis” diagnoses, where eczema encompasses various inflammatory skin conditions while atopic dermatitis refers to chronic, relapsing forms associated with atopic sensitization.^172^ These definitional differences affect microbiome association interpretations and cross-study comparisons, particularly when examining IgE-associated versus non-IgE-associated eczematous conditions.^155^

The evidence supports gut–skin axis involvement in dermatological disease pathophysiology, though population-specific variations and methodological differences highlight the need for standardized approaches, longitudinal studies, and integration of multi-omics data to clarify causal relationships and therapeutic opportunities.

## 5. Gut microbiota dysbiosis as a possible shared mechanism linking neuropsychiatric disorders and their dermatological comorbidities

The evidence presented in the preceding sections reveals the central role of gut microbiota dysbiosis in both neuropsychiatric disorders and skin conditions. What remains largely unexplored, however, is whether gut microbiota dysbiosis could serve as a unifying factor underlying the frequent co-occurrence of these disorders as comorbidities. The emerging hypothesis that gut microbiota dysbiosis simultaneously contributes to both neuropsychiatric and dermatological conditions within the same patient has yet to be thoroughly investigated. Although cohort data are heterogeneous and sometimes contradictory, this novel perspective could offer a comprehensive understanding of how gut microbial alterations influence multiple organ systems and the pathophysiology of these complex conditions.

**Figure 3.**
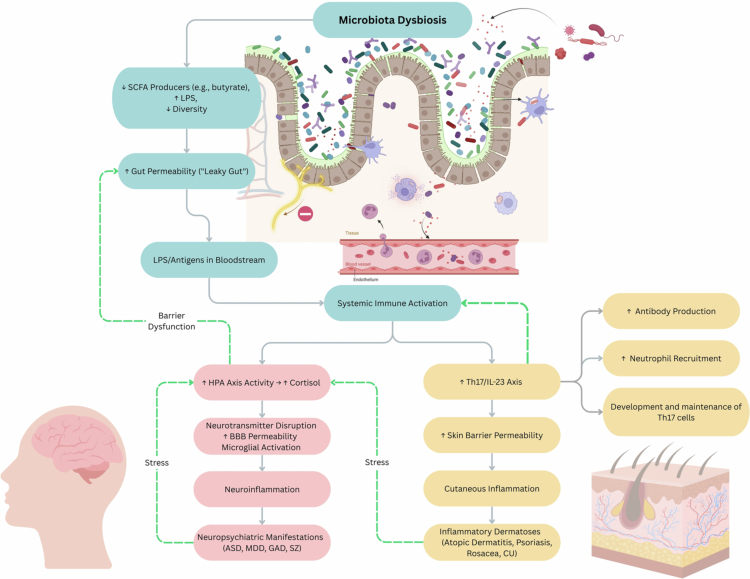
Gut–brain–skin axis: shared pathways linking microbiota dysbiosis to neuropsychiatric and dermatological inflammation. This integrative model illustrates how microbiota dysbiosis triggers a cascade of immune and neuroendocrine changes that contribute to both neuropsychiatric and inflammatory skin conditions through the gut–brain–skin axis. The microbial imbalance promotes loss of SCFA-producing taxa, increased LPS, and reduced microbial diversity, further compromising intestinal barrier function. This compromise in the intestinal defense allows bacterial and antigen translocation to the systemic circulation. The resulting systemic immune activation propagates through two different but interconnected arms: Gut–Brain axis (left): HPA axis hyperactivation, neurotransmitter disruption, and microglial activation promote neuroinflammation, contributing to autism spectrum disorder (ASD), major depressive disorder (MDD), generalized anxiety disorder (GAD), and schizophrenia (SZ). Gut–Skin axis (right): Th17/IL-23-driven immune responses increase skin permeability and inflammation, leading to dermatoses such as atopic dermatitis, psoriasis, rosacea, and chronic urticaria (CU). Green dashed arrows denote indirect positive feedback loops that amplify pathology across the gut–brain–skin network. Neuropsychiatric conditions contributing to central stress and cortisol release further impair gut barrier integrity. Inflammatory skin conditions contribute stress back into the HPA axis, and cutaneous cytokine signaling reinforces systemic inflammatory processes. These feedback loops collectively sustain chronic immune and barrier dysfunction across organ systems. Created in BioRender. Hawkins, B. (2025) https://BioRender.com/adla0kf. Final figure graphics made in Canva.

By analyzing shared compositional and functional microbial patterns across these disorder domains, we propose that these conditions do not represent entirely independent pathological processes. Instead, they appear to converge on fundamental microbial signatures, metabolic disruptions, and immune pathways that collectively support the concept of a gut–brain–skin axis (Figure 3). This synthesis, integrating findings from Tables 1 and 2, highlights gut microbiota dysbiosis as a unifying biological mechanism linking conditions that may seem distinct yet frequently co-occur.

### 5.1. Shared microbial diversity and taxonomic alterations

Reduced microbial diversity emerges as a consistent finding across both neuropsychiatric and skin disorders in human studies. Reduced alpha diversity tracks with depressive symptom burden in large cohort data, while SZ and chronic skin conditions show heterogeneous alpha‑diversity findings alongside consistent compositional and functional dysbiosis, particularly reduced SCFA‑producing taxa.^110^^,^^114^^,^^146^^,^^147^ This parallel reduction in microbial richness suggests a shared underlying mechanism where ecological simplification of the gut microbiome compromises immune homeostasis across organ systems. In contrast, some anxiety subtypes and specific ASD cohorts display increased phylogenetic diversity, though with marked compositional shifts in key bacterial taxa.^118^^,^^121^ Clinical studies in toddlers have shown that higher surgency/extraversion temperament traits are associated with greater phylogenetic diversity, with specific differences in *Dialister*, Rikenellaceae, Ruminococcaceae, and *Parabacteroides* abundance.^84^ This apparent contradiction likely reflects the complex, multifactorial nature of microbiota alterations, where factors such as sampling methodologies, demographic variables, and disease heterogeneity contribute to observed differences, while underlying functional disruptions remain consistent.

The most noticeable shared feature across both neuropsychiatric and dermatological conditions is the consistent depletion of SCFA-producing bacteria in human clinical studies. Families such as Lachnospiraceae and Ruminococcaceae show parallel reductions in ASD, SZ, MDD, atopic dermatitis, and psoriasis.^101^^,^^139^^,^^141^ Consistent with family‑level patterns, cohorts with depression or anxiety often show lower butyrate‑producing genera (e.g., *Coprococcus*/Subdoligranulum) with symptom severity; in GAD vs MDD, *Faecalibacterium* is lower in GAD, aligning with an SCFA‑loss axis.^106^^,^^118^^,^^119^^,^^143^ Preclinical CSU work demonstrates that *Roseburia hominis* supplementation restores SCFA and dampens mast-cell-mediated skin inflammation.^147^

As illustrated in Figure 3, the depletion of beneficial SCFA-producing taxa represents a critical early step in the pathogenic cascade that links gut microbiota dysbiosis to both neuropsychiatric and dermatological manifestations. The loss of these protective bacteria compromises intestinal barrier integrity and reduces the production of anti-inflammatory metabolites that normally maintain immune homeostasis across the gut–brain–skin axis. The functional significance of this shared depletion is supported by preclinical studies, where supplementation with *Roseburia hominis* in a mouse model of CSU markedly increased gut SCFA levels and significantly ameliorated urticaria-like inflammation.^147^ Additionally, the *Roseburia hominis* supplementation reduced vascular permeability, decreased mast cell degranulation, lowered skin mast cell populations, and reduced inflammatory mediators in both skin tissue and plasma.^147^

Parallel to SCFA depletion, both neuropsychiatric and dermatological conditions show enrichment of potentially pro-inflammatory taxa in human studies. *Bacteroides* is enriched in MDD with greater symptom severity and is associated with GAD and anxiety burden (including a strong *Bacteroides*/fiber correlation),^115^^,^^121^ with increases being reported in some ASD cohorts as well.^104^ In adult atopic dermatitis, however, the direction of change varies across studies, so no uniform claim can be made without cohort-specific data.^141^ By comparison, *Sutterella* is higher in GAD than in MDD (while MDD shows lower *Sutterella* than healthy controls), and small biopsy studies have reported increased *Sutterella* in ASD mucosa; suggesting its involvement in shared inflammatory pathways.^106^^,^^118^^,^^141^ The pathogenic role of specific pro-inflammatory taxa is supported by experimental evidence showing that gavage with *Klebsiella pneumoniae* in mice significantly increased urticaria-like inflammation, enhanced mast cell activation, and raised systemic Lipopolysaccharide (LPS) levels through TLR4-mediated mechanisms.^147^

### 5.2. Pathophysiological model and feedback loops

The mechanistic framework depicted in Figure 3 illustrates how the initial microbial imbalance, characterized by loss of SCFA producers, increased LPS-producing bacteria, and reduced diversity, triggers a cascade of interconnected pathological processes. This dysbiotic state promotes bacterial and antigenic translocation into systemic circulation, initiating immune activation that propagates through two interconnected pathways: the gut–brain axis and the gut–skin axis.

In the gut–brain pathway, systemic immune activation leads to HPA axis hyperactivation, neurotransmitter disruption, and microglial activation that collectively promote neuroinflammation. This pathway contributes to the pathogenesis of ASD, SZ, MDD, and GAD, as evidenced by the consistent elevations in pro-inflammatory cytokines and dysregulated cortisol patterns documented across these conditions in human studies.^34^^,^^52^^,^^63^^,^^173^

Simultaneously, the gut–skin arm involves Th17/IL-23-driven immune responses that increase skin permeability and inflammation, leading to dermatoses such as atopic dermatitis, CU, psoriasis, and rosacea. The elevation of IL-17 and IL-23, as documented in both skin conditions and neuropsychiatric disorders, supports this shared inflammatory pathway.^63^^,^^74^

A critical aspect of the model shown in Figure 3 is the presence of positive feedback loops that amplify pathology across the gut–brain–skin network. Clinical evidence suggests that neuropsychiatric conditions contribute to central stress and cortisol release,^174^ which further impairs gut barrier integrity.^175^^,^^176^ Conversely, inflammatory skin conditions feed stress back into the HPA axis,^177^ while cutaneous cytokine signaling reinforces systemic inflammatory processes.^178^^,^^179^ These bidirectional interactions help explain why neuropsychiatric and dermatological comorbidities tend to persist and exacerbate each other over time.

### 5.3. Metabolic and immune-inflammatory pathways

Reduced SCFA production represents a key mechanistic link between gut dysbiosis and dual-organ pathology. Human metabolomic studies indicate shared functional disturbances across neuropsychiatric and dermatologic disorders, with several cohorts showing lower levels of SCFA, particularly butyrate, propionate, and acetate, in depression, anxiety, and SZ, as well as in CSU among skin conditions.^118^^,^^147^^,^^180^ The therapeutic relevance of SCFA restoration is demonstrated by preclinical studies showing that oral administration of caproate significantly suppressed mast cell activation, reduced vascular permeability, and lowered pro-inflammatory cytokine expression in skin tissue in mice.^147^

Amino acid metabolism shows parallel disruptions across neuropsychiatric and dermatological conditions in human studies. In ASD, multi-omic and interventional work reports alterations in amino acid–related metabolism and predicted microbial functions rather than consistent elevations of stool branched-chain amino acids. In GAD, Mendelian randomization studies suggest causal links between specific gut taxa and anxiety risk, but do not show pathway-level enrichment for branched-chain amino acid biosynthesis.^105^^,^^120^ SZ, in contrast, show enrichment of glutamate, methionine, and tryptophan catabolism pathways, while dermatological conditions show altered histamine and tryptophan metabolism.^147^^,^^180^ Collectively, these shared metabolic alterations suggest common disruptions in neuromodulatory balance and inflammatory signaling, with the glutamate-GABA imbalance observed in SZ paralleling the disrupted amino acid pools seen in chronic skin conditions.

The immune landscape depicted in Figure 3 reveals striking parallels between neuropsychiatric and dermatological conditions in human studies, with four cytokines consistently elevated across both domains: IL-6, TNF-*α*, IL-1β, and IL-17. This shared inflammatory profile supports the gut–brain–skin axis model, illustrating how these cytokines link gut dysbiosis to pathology in distant organs. IL-6 exemplifies this convergence most clearly, tracking with depressive severity and anxiety symptoms in neuropsychiatric conditions while simultaneously sustaining keratinocyte activation in psoriatic plaques.^52^^,^^63^ TNF-*α* plays a similar dual role, orchestrating immune cell trafficking in psoriatic skin and correlating with neuroinflammatory changes and depressive symptom burden.^59^ IL-1β triggers inflammatory cascades in both atopic dermatitis and CU while contributing to immune dysregulation in MDD.^51^^,^^74^ IL-17, the hallmark effector in psoriasis, is also elevated in GAD and SZ, indicating Th17-skewed immunity across both domains.^63^^,^^64^

The barrier dysfunction component of Figure 3 illustrates how reduced SCFA production compromises intestinal tight junctions, permitting bacterial translocation and LPS entry into systemic circulation. This microbial translocation triggers immune activation that reaches both brain and skin, contributing to neuroinflammation and cutaneous inflammation simultaneously. Experimental evidence comes from preclinical studies: fecal microbiota transplantation from CU patients to mice increased intestinal permeability and downregulated barrier function markers.^147^ Additionally, studies using NC/Nga mice showed that *Bifidobacterium bifidum* interventions affected T cell differentiation and gut microbiota composition, providing further insights into the gut–skin axis in atopic dermatitis pathogenesis.^181^

### 5.4. Clinical implications and therapeutic convergence

The integrated model presented in Figure 3 has direct clinical implications, suggesting that interventions targeting any component of the gut–brain–skin axis may provide benefits across multiple organ systems. Clinical studies report that interventions targeting gut microbiota restoration, including specific probiotic strains, SCFA supplementation, and anti-inflammatory approaches, show efficacy across both domains. Preclinical studies support these clinical observations, with animal models showing that antibiotic eradication of pathogenic *Klebsiella pneumoniae* reduced urticaria-like inflammatory responses, while *Roseburia hominis* and SCFA supplementation provided protective effects against mast cell-mediated inflammation.^147^

The recognition that cytokine-targeting biologics may benefit both dermatological inflammation and psychiatric comorbidities, as suggested by the shared inflammatory pathways in Figure 3, further supports the clinical relevance of this integrated approach. Studies showing simultaneous improvement in both mood and skin lesions following microbiota-targeted interventions provide compelling evidence for therapeutic strategies that address the gut–brain–skin axis as a unified system.

### 5.5. Synthesis-driven implications

By synthesizing evidence across neuropsychiatric and dermatological conditions within the model illustrated in Figure 3, this review establishes gut microbiota dysbiosis as a central biological interface linking brain and skin health. The shared pathways of SCFA depletion, pro-inflammatory taxa enrichment, cytokine elevation, and barrier dysfunction provide a blueprint for understanding the frequent comorbidity between these conditions. Clinical studies substantiate these patterns in human patients, while preclinical research clarifies how specific microbial alterations drive pathology across organ systems. The positive feedback loops depicted in Figure 3 explain how these comorbidities become self-sustaining and mutually reinforcing over time. This integrated understanding positions the gut microbiota as both a diagnostic biomarker and therapeutic target for patients presenting with complex neuropsychiatric-dermatological comorbidities.

Rather than treating these conditions as separate entities, the gut–brain–skin axis suggests that targeting microbial restoration may simultaneously ameliorate symptoms across both disease domains, offering precision strategies for complex comorbidity patterns. The collected evidence supports a need for a novel shift toward integrated care models that recognize the gut microbiota's central role in linking mental health and skin conditions, opening new doors for both understanding disease mechanisms and developing targeted therapeutic interventions.

## 6. Therapeutic approaches: Gut microbiota-based interventions

The significance of gut microbiota dysbiosis at the interface between neuropsychiatric disorders and their dermatological comorbidities extends beyond mechanistic understanding to therapeutic potential. This unified approach could lead to treatments addressing both neuropsychiatric symptoms and skin conditions simultaneously. Therefore, this section focuses on emerging therapeutic strategies targeting gut microbiota that alleviate neuropsychiatric disorders and/or dermatological conditions. Growing evidence suggests that gut microbiota dysbiosis plays an active, causative role in pathogenesis. By modulating composition and metabolic activity through probiotics, prebiotics and/or dietary modifications, interventions show promise in restoring microbial balance, reducing systemic inflammation, and improving both mental health outcomes and skin pathology.

### 6.1. Gut microbiota-targeted therapies for neuropsychiatric disorders

Interventions targeting gut microbiota have shown promising effects on behavioral and immune-related symptoms in ASD, with randomized, double-blind trials providing the strongest evidence for probiotic efficacy. In an eight-child crossover pilot, colostrum-only treatment produced significant drops in Aberrant Behavior Checklist irritability, stereotypy, and hyperactivity, with reduced helper T cells and IL-13 in combination treatment.^182^ A Taiwanese study randomizing 80 boys to *Lactobacillus plantarum* PS128 showed 10–13% reductions in SNAP-IV hyperactivity and oppositional-defiance scores.^183^ Similarly, an eight-strain De Simone formulation shifted frontopolar β/γ power, correlating with fewer repetitive behaviors and lower plasma TNF-*α* in preschoolers.^184^ Building on these clinical trial findings, non-randomized/observational studies demonstrate real-world efficacy and strain-specific performance. An Italian cohort showed that 86.7% of children receiving PS128 improved on Clinical Global Impression versus 38.5% using assorted probiotics.^185^ Extending this research, another study found significant Clinical Global Impression improvements with probiotic-oxytocin combination alongside gut microbiome changes.^186^ In parallel investigations, *Limosilactobacillus reuteri* lowered Social Responsiveness Scale scores and raised Adaptive Behavior Assessment System-2 scores.^187^

Further biological insights emerge from exploratory studies examining microbiota-behavior relationships. Additional studies showed that synbiotic-induced microbiota blooms reduced autism rating scores.^188^^,^^189^ Small-scale studies suggest that the probiotic VSL#3 may improve social behavior in individuals with ASD,^190^ who show differences in Bacteroidetes/Firmicutes ratios and immune markers^92^ and reduced urinary D-arabinitol.^191^ Reinforcing these intervention findings, cross-sectional profiling links altered SCFA and lysozyme to ASD severity.^192^

Transitioning to SZ, probiotics address both metabolic and psychiatric symptoms associated with SZ. Combined probiotic-fiber interventions effectively limited weight gain and improved BMI and fasting insulin,^193^^,^^194^ while probiotics alone showed only transient effects,^195^ emphasizing the importance of synergistic approaches. Regarding psychiatric symptom modulation, studies reveal mixed but informative results. Synbiotics reduced IL−6 and accelerated Positive and Negative Syndrome Scale (PANSS) improvements during risperidone therapy,^196^ while *Bifidobacterium breve* A−1 improved Hospital Anxiety and Depression Scale scores^197^ in a study on mental well-being. Additional cognitive and metabolic benefits included enhanced performance and cholesterol reduction,^198^ and improved antioxidant capacity.^199^ However, providing important context for probiotic limitations, large trials with *Lactobacillus rhamnosus* GG plus *Bifidobacterium animalis* showed no PANSS changes,^200^^,^^201^ though targeting the mycobiome reduced *Candida albicans* IgG in a preclinical rat study.^202^

For depressive disorders, clinical trials increasingly support probiotic efficacy in MDD through multiple therapeutic pathways. A study found *Lactobacillus helveticus* R0052 and *Bifidobacterium longum* R0175 significantly reduced Beck Depression Inventory scores.^203^ Similarly, Akkasheh et al. demonstrated Beck Depression Inventory score reductions with multi-strain probiotics, while Schaub et al. showed Hamilton Depression Rating Scale improvements with Vivomixx.^204^^,^^205^ To explain these clinical benefits, probiotics may modulate tryptophan metabolism, the sole precursor for serotonin, influencing serotonergic signaling via intestinal enterochromaffin cells and mechanosensitive Piezo2 pathways.^206^ In preclinical studies, supplementation with *Bifidobacterium breve* CCFM1025 was shown to reduce 5-HT turnover,^207^ with another study reporting decreased kynurenine/tryptophan ratios.^203^ Notably, even without immediate mood effects, synbiotics increased butyrate and suppressed IL-6, suggesting gut–brain pathway priming requiring longer treatment.^208^ Clinically, a pilot RCT showed greater depressive improvement with probiotics versus placebo over 8 weeks,^209^ and multiple meta-analyses report small-to-moderate effects in depression and anxiety, varying by strain.^210^

Expanding beyond depressive disorders to anxiety disorders, multi-strain probiotics show emerging therapeutic potential for GAD/anxiety through modulation of stress response pathways. One study reported reduced Hamilton Anxiety scores and a halved anxiety threshold with high-dose multi-strain “psychobiotics”,^211^ while another found decreased stress and anxiety after a 28-day multi-strain intervention.^212^ However, not all studies demonstrate consistent subjective improvements; *Lactobacillus casei* strain Shirota prevented exam-related cortisol spikes without reducing self-reported anxiety, suggesting HPA-axis modulation may precede behavioral changes.^213^ Preclinical mouse studies support this mechanism: *Lactobacillus plantarum* JYLP-326 reversed test-induced gut dysbiosis, highlighting bidirectional stress–microbiota interactions.^214^

### 6.2. Gut microbiota-targeted therapies for dermatological conditions

Shifting focus from neuropsychiatric to dermatological applications, limited research reveals promising probiotic potential for skin conditions (summarized in Table 3). Probiotics demonstrate consistent benefits in atopic dermatitis, particularly in pediatric populations, through immune modulation. Preclinical studies established biological foundations, showing that *Lactobacillus paracasei* NTU 101 reduces skin inflammation and promotes tolerogenic immune responses in mouse models.^215^ Translating these findings to clinical settings, several studies confirmed therapeutic efficacy. Microbiota changes were associated with SCORAD improvements,^157^ and one trial reported an 83% SCORAD reduction versus 24% with placebo.^156^ In pediatric populations, *L. rhamnosus* GG reduced SCORAD scores and improved quality of life.^158^ Early intervention studies further demonstrate strain-specific preventive effects: *L. rhamnosus* HN001 administered from pregnancy through infancy significantly reduced eczema prevalence, though effects on atopic sensitization were limited, suggesting targeted rather than broad immune modulation.^170^

Turning to seborrheic dermatitis, emerging evidence suggests therapeutic potential through antimicrobial and immunomodulatory actions. Metabolites of *Bifidobacterium lactis* and *Lacticaseibacillus rhamnosus* demonstrated over 90% growth inhibition of *Malassezia furfur* and *Cutibacterium acnes*, providing a mechanistic rationale for clinical applications.^216^ Translating these preclinical findings to humans, supplementation with *Lactobacillus paracasei* ST11 significantly improved dandruff, erythema, and seborrhea by suppressing Th1/Th2 cytokines and upregulating IL-10/TGF-*β.*^217^

For CSU, as one of the most challenging skin conditions to treat, multi-strain probiotics combined with antihistamines were shown to significantly reduce UAS7 scores, likely through gut–skin axis modulation involving increased TH1 cytokines and reduced IgE.^161^ Similarly, a Yimingjia probiotic blend was reported to reduce wheal size and attack frequency.^159^ Providing support for these therapeutic effects, gut microbiota dysbiosis with reduced beneficial microbes has been reported in CSU patients, suggesting probiotics may help counter impaired mucosal tolerance.^218^ Further validating this approach, synbiotic combinations were shown to provide greater symptom reductions than antihistamines alone, consistent with TLR activation and microbiota restoration principles.^159–161,218^

For psoriasis, the most extensively studied dermatological condition for probiotic intervention, research consistently supports their promise as an adjunctive therapy. Preclinical studies have shown symptom amelioration with specific strains that reduce pro-inflammatory cytokines.^219^ Clinical findings indicate that multi-strain approaches outperform single-strain interventions, with one trial reporting a 75% Psoriasis Area and Severity Index (PASI) reduction in 66.7% of patients,^163^ and a recent review summarizing marked PASI and Dermatology Life Quality Index improvements alongside reductions in inflammatory biomarkers.^166^ Single-strain studies show more modest benefits,^164^^,^^165^ emphasizing formulation complexity. Probiotics also exert systemic effects, reducing markers such as CRP and TNF-*α*^162^, modulating cytokines, and enriching beneficial microbial taxa.^166^

In the case of rosacea, research remains limited but shows emerging therapeutic potential. Preclinical studies have observed that *Ligilactobacillus salivarius* 23−006 and *Lacticaseibacillus paracasei* 23−008 may improve symptoms through TLR2/MyD88/NF-κB pathway modulation and microbiota restoration.^220^ Clinically, multi-strain probiotics combined with doxycycline improved symptoms, reduced inflammatory markers, and enhanced skin hydration.^168^ Sustained therapeutic benefits have also been reported, with maintained scalp rosacea remission under combined treatment.^167^ However, highlighting strain specificity, a recent trial found no significant benefits with a single-strain intervention, reinforcing the need for a patient-specific approach similar to insights gained in psoriasis research.^169^

Collectively, these studies demonstrate that gut microbiota modulation holds promise for both neuropsychiatric and dermatological conditions, supporting the gut–brain–skin axis concept. Multi-strain formulations generally outperform single-strain approaches, and combination therapies show enhanced efficacy. These patterns suggest shared mechanisms that could inform integrated treatment strategies. Future research should focus on strain-specific effects, optimal dosing, and therapies targeting both neuropsychiatric and dermatological manifestations simultaneously.

## 7. Future perspectives: Can targeting the gut microbiota simultaneously improve neuropsychiatric conditions and their comorbid skin disorders?

An emerging insight from this review is the therapeutic potential of targeting the gut microbiota, particularly *Lactobacillus* species, to treat both neuropsychiatric disorders' core phenotypes and their associated dermatological comorbidities. Preclinical studies have shown that *Lactobacillus* supplementation can reduce inflammatory cytokines linked to psoriasis severity^219^^,^^221^ and is among the most abundant genera associated with improved brain function in MDD models.^222^ It also supports skin health by reducing inflammation and enhancing barrier integrity.^223^ Our reviewed clinical trials reinforce this trend: for example, one study reported significant PASI75 responses using a formulation containing *Lactobacillus rhamnosus,*^163^ while another study found marked SCORAD score improvements in atopic dermatitis with *L. rhamnosus GG.*^158^ These findings highlight the cross-system therapeutic effects of *Lactobacillus*, likely mediated by its anti-inflammatory and microbiota-modulating properties.

Several major limitations must be addressed to advance this promising field. The variable outcomes across studies underscore critical gaps that must be addressed before unified treatments can be developed. Our review focused on specific neuropsychiatric disorders and their most consistently documented dermatologic comorbidities to maintain clarity, but this necessarily excluded many potentially relevant conditions. The disparity between successful multi-strain interventions (e.g.,^166^) and weaker single-strain outcomes (e.g.,^165^) illustrates the complexity of microbiota-based therapies. This reflects a broader limitation in the field: few psychiatric or dermatological disorders have been systematically studied through the gut–brain–skin axis framework. Broadening the scope could uncover shared microbial patterns and mechanisms, enabling more unified therapeutic strategies. Another major limitation lies in the short duration and cross-sectional nature of most studies. Trials like those by^166^ and^163^ typically last 8–12 weeks, restricting insight into long-term effects. While some sustained benefits, such as reduced relapse rates reported by Navarro-López et al., during six-month follow-ups,^163^ suggest therapeutic promise, long-term longitudinal studies are essential to understand the temporal dynamics of dysbiosis. These studies should clarify whether microbial shifts precede symptom onset, occur alongside disease progression, or are secondary to external influences like medication, diet, or stress. Inconsistencies across studies are also amplified by the lack of control for known microbiome-influencing variables, including age, geography, circadian rhythms, and medication. This reduces cross-study comparability and may explain inconsistent findings. For instance, pediatric-focused studies on atopic dermatitis (e.g.,^157^^,^^158^) may not generalize to adult populations, as microbiota composition changes with age.^157^ While animal models offer better control of confounders, human studies must address these complexities through well-designed, diverse, and longer-term trials. Biological differences such as sex, hormonal status, and life stage also influence microbiota composition and host response. For example, estrogen enhances microbial richness and shifts gut microbial profiles,^224^ potentially contributing to sex-specific disease patterns or treatment responses. Yet, few studies stratify participants by sex or hormonal status, potentially obscuring relevant mechanisms. The baseline microbiome variability described by Huang et al.,^194^ where increased richness predicted lower weight gain in SZ patients, suggests that personalized microbiome analysis could improve outcomes.

In summary, while there is growing evidence supporting gut microbiota's role in both neuropsychiatric and dermatologic health, targeting it for dual benefit remains an underexplored but promising avenue. To realize this potential, future research must move beyond narrow scopes and short-term trials. Longitudinal, inclusive, and stratified studies are needed to better define the gut–brain–skin axis and guide precision probiotic therapies that offer consistent benefits across interconnected conditions.

## 8. Conclusion

This review brings together emerging data from several fields to suggest a possible biological interface between neuropsychiatric disorders and their skin comorbidities, with gut microbial imbalance as a central feature. Across multiple conditions, including autism spectrum disorder, schizophrenia, depression, and generalized anxiety disorder, depression, similar microbial alterations appear again in dermatologic conditions like atopic dermatitis, chronic urticaria, psoriasis, and rosacea. These shared patterns include fluctuations in microbial diversity, loss of short-chain fatty acid producers, and increased abundance of pro-inflammatory taxa. The recurrence of these findings across independent studies suggests an underlying connection that warrants further exploration. Although causality cannot yet be confirmed, the consistency of these findings across independent studies supports the idea that the gut microbiota dysbiosis may play a key role in shared pathological processes. By synthesizing results from clinical and preclinical research, this review outlines a framework for understanding how immune, metabolic, and neural signaling pathways could interact through the gut–brain–skin axis. Rather than approaching neuropsychiatric and skin symptoms as entirely separate phenomena, it may be beneficial to consider their shared biological features when designing future studies. Targeting gut microbiota dysbiosis in shared therapeutic strategies could provide a more comprehensive approach to treating both disorder domains simultaneously.
